# The Relationship Between Perceived Organizational Justice and Workplace Alienation Among Nurses in Northern Saudi Arabia: A Cross‐Sectional Study

**DOI:** 10.1002/hsr2.73420

**Published:** 2026-10-26

**Authors:** Ahmad Rayan, Mamdooh Arrowaili, Manal Hassan Baqeas, Fadwa Alhalaiqa, Suhair Al‐Ghabeesh, Hanan Alyami, Mirna Fawaz

**Affiliations:** ^1^ Psychiatric and Mental Health Nursing, and Dean Zarqa University Zarqa Jordan; ^2^ Faculty of Nursing Zarqa University Zarqa Jordan; ^3^ College of Nursing Northern Border University Arar Saudi Arabia; ^4^ Professor/Psychiatric and Mental Health Nursing Freelancer; ^5^ Al‐Zaytoonah University of Jordan, Faculty of Nursing. Airport Street Amman Jordan; ^6^ Department of Medical and Surgical Nursing Princess Norah Bint Abdurrahman University, College of Nursing Riyadh Saudi Arabia; ^7^ Nursing Department Beirut Arab University, Faculty of Health Sciences Beirut Lebanon

**Keywords:** nurses, perceived organizational justice, Saudi Arabia, workplace alienation

## Abstract

**Background and Aims:**

Perceived organizational justice may influence nurses' workplace alienation. This study examined the relationship between perceived organizational justice and workplace alienation among nurses in Northern Saudi Arabia.

**Methods:**

A descriptive cross‐sectional study was conducted among 183 nurses working in government hospitals in the Al‐Jouf region, Saudi Arabia. Participants completed the Colquitt Organizational Justice Scale and the Work Alienation Scale. Data were analyzed using descriptive statistics, Pearson correlation, group‐comparison tests, and simple linear regression.

**Results:**

Mean perceived organizational justice was 4.04 (SD, 0.39) and mean workplace alienation was 2.71 (SD, 0.47), each scored from 1 to 5. Perceived organizational justice was inversely associated with workplace alienation (r=−0.455, p<0.001). Perceived organizational justice explained 20.7% of the variance in workplace alienation ($R^{2}=0.207$, p<0.001).

**Conclusion:**

Higher perceived organizational justice was associated with lower workplace alienation among nurses. Longitudinal research is needed to examine temporal and causal relationships.

## 1 Introduction

Saudi Arabia's Vision 2030 has made healthcare reform a national priority through workforce development, privatization, digitalization, and performance improvement [1]. Nurses, as frontline healthcare providers, face job dissatisfaction, increasing workloads, and rapidly changing healthcare demands [2, 3]. These pressures may contribute to perceptions of organizational injustice, including unfair resource distribution, inconsistent decision‐making, poor interpersonal treatment, and inadequate communication, which can undermine nurses' well‐being, engagement, and performance [4].

Perceived organizational justice refers to employees' perceptions of fairness in the workplace and includes distributive, procedural, interpersonal, and informational justice [5]. Among nurses, greater perceived organizational justice is associated with lower emotional exhaustion and better job satisfaction and well‐being [6]. In contrast, workplace alienation involves powerlessness, meaninglessness, and disconnection from work and is linked to adverse work‐related outcomes [7, 8].

Among Saudi nurses, hierarchical structures, centralized decision‐making, limited autonomy, and workplace cultural factors may increase work‐related pressures, particularly in northern regions. Organizational justice is not the only determinant of workplace alienation; however, examining perceptions of fairness provides a focused way to assess its association with nurses' work experiences. This issue is especially relevant in government hospitals in Northern Saudi Arabia, where healthcare reforms may not fully address perceived inequities in compensation, resource allocation, task distribution, participation in decision‐making, and interpersonal treatment [9]. Such perceptions may undermine nurses' workplace experiences and contribute to negative outcomes, including workplace bullying and alienation [7, 10, 11]. Limited Saudi‐specific evidence from northern regions also constrains context‐sensitive strategies to support nurse retention and quality care [12].

Addressing this gap may benefit nurses, healthcare managers, policymakers, researchers, and patients. Understanding the association between organizational justice and workplace alienation may help healthcare organizations develop fairer work environments that support nurses' well‐being, job satisfaction, and retention. Managers may use this evidence to improve communication, decision‐making, workload distribution, and staff‐development initiatives. In line with Vision 2030, policymakers may consider organizational‐justice indicators when evaluating healthcare workplaces. Ultimately, strengthening organizational fairness may support nurse retention, quality of care, patient safety, and compassionate service delivery.

This descriptive cross‐sectional study examines the levels of perceived organizational justice and workplace alienation among nurses in Northern Saudi Arabia's government hospitals, their interrelationship, and the predictive roles of justice and sociodemographic/work‐related factors on alienation. Specific objectives are: (1) to explore levels of perceived organizational justice (distributive, procedural, interpersonal, informational) and workplace alienation (self‐estrangement, powerlessness, meaninglessness); (2) to identify their correlation; and (3) to determine predictive contributions.

### 1.1 Research Questions

1. What are the levels of perceived organizational justice and workplace alienation among nurses in Northern Saudi Arabia?
2. What is the relationship between perceived organizational justice and workplace alienation?
3. To what extent do perceived organizational justice, sociodemographic characteristics, and work‐related factors predict workplace alienation?
4. Is there a significant relationship between nurses' sociodemographic characteristics and the main study variables?

## 2 Methodology

### 2.1 Research Design

The descriptive correlational methodology that was employed in this study included the cross‐sectional design. This cross‐sectional study is reported in accordance with the Strengthening the Reporting of Observational Studies in Epidemiology (STROBE) guideline.

### 2.2 Setting

This study was conducted in government hospitals within the Al‐Jouf Health Cluster, Saudi Arabia, a region undergoing healthcare transformation under Saudi Vision 2030, emphasizing governance, accountability, efficiency, and patient safety [13]. These hospitals operate under Ministry of Health governance with centralized structures, hierarchical leadership, and formal administrative and performance‐evaluation systems [14]. The nursing workforce is diverse, comprising Saudi and expatriate nurses with varied educational and clinical backgrounds.

### 2.3 Population and Sample

The target population comprised registered nurses working in government hospitals within the Al‐Jouf Health Cluster in northern Saudi Arabia. The study included nurses from Prince Mutaib Hospital (343 beds; 343 nurses; 16 departments), Al‐Qurayat General Hospital (310 beds; 340 nurses; 16 departments), and King Abdulaziz Specialist Hospital (300 beds; 285 nurses; 18 departments). These hospitals provided a diverse sample of nurses working within the region's healthcare governance system.

### 2.4 Sampling

Convenience sampling was used to recruit registered nurses directly involved in clinical care. Nurses in training, temporary‐contract nurses, administrative nurses, and non‐Saudi nurses were excluded. Sample size was calculated using G*Power 3.1 for multiple linear regression with seven predictors, a medium effect size (*f* ^2^ = 0.15), *α* = 0.05, and 95% power, yielding a minimum of 168 participants [15]. Allowing for 10% potential nonresponse or incomplete data, the target sample was 185 nurses. Of the 185 nurses invited, 183 responded, yielding a 98.9% response rate.

### 2.5 Ethical Considerations

Ethical approval was obtained from the Institutional Review Board of the Faculty of Nursing, Zarqa University (IRB No. 84/2024/2025). Participants received information about the study purpose, procedures, potential risks and benefits, and their rights before providing written informed consent. Participation was voluntary, and withdrawal involved no penalty or loss of benefits. Confidentiality and anonymity were maintained by excluding personal identifiers and assigning each questionnaire a unique code. Data were securely stored and accessible only to the principal investigator and research supervisor and were used solely for academic and scientific purposes. The study adhered to the ethical principles of the Declaration of Helsinki.

### 2.6 Measurement

#### 2.6.1 Demographic Variables

In this study, six sociodemographic and work‐related variables were relevant. These variables include gender, age category, educational level, marital status, work area or unit in this hospital and years of experience as a nurse. These variables were identified from previous relevant studies.

#### 2.6.2 Perceived Organizational Justice

A Colquitt Organizational Justice Scale (2001) was used to measure variable, consists of 20 items covering 4 dimensions: procedural justice (7 items), distributive justice (4 items), interpersonal justice (4 items), and informational justice (5 items), with higher scores indicating greater perceptions of fairness. Responses range from 1 (Strongly Disagree) to 5 (Strongly Agree). Higher scores indicate greater perceived justice. This instrument helped assess nurses' perceptions of fairness in procedures, outcomes, personal treatment, and communication within their hospitals. To assess internal consistency and ensure the reliability of the questionnaire, previous studies reported varying Cronbach's alpha values, with a minimum of 0.70 for all reliability criteria [16]. For example, Song et al. (2024) reported a Cronbach's alpha of 0.91, and Zahran and Eldossoqi [17], reported a Cronbach's alpha of 0.909, this indicating high internal consistency.

#### 2.6.3 Workplace Alienation

To measure workplace alienation, the 18‐item Work Alienation Scale (WAS [18]) was employed. This scale assesses three core dimensions—self‐estrangement (9 items), powerlessness (4 items), and meaninglessness (5 items)—adapted for contemporary organizational contexts while retaining the original theoretical structure. Items use a 5‐point Likert response format (1 = *Strongly Disagree* to 5 = *Strongly Agree*), where higher composite scores indicate greater work alienation.

The scale demonstrates robust psychometric properties. Gümüş and Taş (2021) confirmed *α* = 0.88 (total) and 0.69–0.78 (subscales) in nursing samples. Construct validity is supported through exploratory and confirmatory factor analyses maintaining the three‐factor solution [18], with convergent validity evidenced by significant correlations with burnout (*r* = 0.45–0.62) and job dissatisfaction (*r* = 0.50) in healthcare settings (Gümüş & Taş 2021).

#### 2.6.4 Data Collection Procedure

Following IRB approval from Zarqa University and formal approval from the Saudi Ministry of Health and participating hospitals, electronic invitations were distributed through hospital email, internal WhatsApp groups, and nursing department portals. The Google Form included the study information sheet, electronic informed consent, and sections on sociodemographic/work‐related characteristics, the Colquitt Organizational Justice Scale, and the Work Alienation Scale. Completion required approximately 15–20 min.

Data were collected over 4 weeks from June to July 2025. Google Forms was configured not to collect email addresses, IP addresses, or other identifying information. Data were securely stored in the principal investigator's account, accessible only to the research team, then exported to Microsoft Excel and analyzed using IBM SPSS Statistics version 26. This online approach supported participant privacy and facilitated data collection across participating hospitals.

### 2.7 Statistical Analysis

Data were exported from Google Forms to Microsoft Excel and analyzed using IBM SPSS Statistics for Windows, version 26.0 (IBM Corp., Armonk, NY, USA). Data were screened for missing values, duplicate records, and data‐entry errors before analysis. Categorical variables were summarized as n (%), and continuous variables were summarized as mean (SD) when approximately normally distributed. Independent‐samples *t*‐tests compared workplace‐alienation scores between 2‐group characteristics; one‐way analysis of variance compared scores across characteristics with 3 or more groups; Pearson correlation assessed associations between continuous variables; and simple linear regression evaluated the association between perceived organizational justice and workplace alienation. All tests were two‐sided, and p<0.05 was considered statistically significant. Effect estimates, including correlation coefficients, regression coefficients, and explained variance, were reported alongside *P* values.

## 3 Results

### 3.1 Sociodemographic and Work‐Related Characteristics

A total of 183 nurses participated in the study. As presented in Table 1, the mean age was 34.89 years (SD = 6.87). Of the 183 participants, 112 (61.2%) were male and 71 (38.8%) were female. In terms of education level, the majority held a Bachelor's degree (*n* = 143, 78.1%). A total of 152 participants (83.1%) were married and 31 (16.9%) were single.

**Table 1:** Sociodemographic and work‐related characteristics of participants.

Variable	M	SD
**Age (years)**	34.89	6.87

Variable	Frequency (*n*)	Percentage (%)
**Gender**		
Male	112	61.2%
Female	71	38.8%
**Educational level**		
Bachelor's	143	78.1%
Master's	40	21.9%
**Marital status**		
Married	152	83.1%
Single	31	16.9%
**Work area or unit in this hospital**		
Medical/Surgical departments	27	14.8%
Emergency department	53	29%
Critical care departments	27	14.8%
OB/GYN departments	27	14.8%
Pediatric departments⁠	20	10.9%
Outpatient departments	16	8.7%
Others	13	7.1%
**Years of experience as a nurse**		
Less than 5 years	29	15.8%
5 to less than 10 years	52	28.4%
10–less than 15 years	47	25.7%
15 years and older	55	30.1%

Abbreviations: M, mean; SD, standard deviation; n, frequency; %, Percentage.

Work‐related characteristics as shown in Table 1, with regard to the variable Work Area or Unit in this Hospital, it became clear that nurses working in the Emergency Department are the most frequent (*n* = 53, 29%), followed by nurses working in each of the Medical/Surgical Departments, Critical Care Departments, and OB/GYN Departments, with a frequency of (27) for each of these three departments and a percentage of (14.8%) for each of them, and finally the participants who chose the option: Others, and their number is (13) nurses and a percentage of (7.1%). Regarding years of experience as a nurse, the numbers of employees were close in the long periods, as the number of nurses with years of experience: 15 years and older (*n* = 55, 30.01%), then nurses with years of experience: 5 to less than 10 years (*n*: 52, 28.4%), followed by nurses with years of experience: 10 to less than 15 years (*n*: 47, 25.7%), and finally nurses with years of experience: less than 5 years (*n*: 29, 15.8%).

### 3.2 The Level of Perceived Organizational Justice

Perceived organizational justice was summarized as a mean score because scores were approximately normally distributed. On the 1‐to‐5 scale, where higher scores indicate greater perceived organizational justice, the mean score was 4.04 (SD, 0.39). The highest was for Distributive Justice (*M* = 4.06, SD = 0.45), followed by Informational Justice (*M* = 4.05, SD = 0.52), Procedural Justice (*M* = 4.03, SD = 0.42), and finally, Personal Justice (*M* = 4.00, SD = 0.46). Workplace alienation was summarized as a mean score because scores were approximately normally distributed. On the 1‐to‐5 scale, where higher scores indicate greater workplace alienation, the mean score was 2.71 (SD, 0.47).

### 3.3 The Level of Workplace Alienation

Workplace alienation had a mean score of 2.71 (SD, 0.47) on a possible 1‐to‐5 scale, with higher scores indicating greater alienation. This mean falls below the scale midpoint of 3, suggesting that participants generally reported relatively low workplace alienation. The arithmetic means for the sub‐dimensions of Workplace Alienation ranged from 3.73 to 2.68. Self‐Estrangement recorded the highest value (*M* = 2.73, SD = 0.48), followed by Powerlessness (*M* = 2.71, SD = 0.57), which had a medium score skewed to low, and finally meaninglessness (*M* = 2.68, SD = 0.52).

### 3.4 The Relationship Between Perceived Organizational Justice and Workplace Alienation

A Pearson Correlation test was conducted to determine the relationship between perceived organizational justice and workplace alienation. Pearson correlation analysis showed a moderate inverse association between perceived organizational justice and workplace alienation (r=−0.46, p<0.01). Thus, nurses who perceived greater organizational justice tended to report lower workplace alienation (Table 2). The study also showed an inverse (negative) and statistically significant relationship between the dimensions of perceived organizational justice and workplace alienation as follows: Procedural justice (*r* = −0.41, p<0.01), Distributive justice (*r* = −0.39, p<0.01), Interpersonal justice (*r* = −0.40, p<0.01), and informational justice (*r* = −0.41, p<0.01).

**Table 2:** The relationship between perceived organizational justice and workplace alienation.

Variables	1	2	3	4	5	6
1. Procedural justice	1.00	0.74**	0.72**	0.76**	0.93**	−0.41**
2. Distributive justice	0.74**	1.00	0.69**	0.66**	0.86**	−0.39**
3. Interpersonal justice	0.72**	0.69**	1.00	0.65**	0.85**	−0.40**
4. Informational justice	0.76**	0.66**	0.65**	1.00	0.87**	−0.41**
5. Perceived organizational justice, overall	0.93**	0.86**	0.85**	0.87**	1.00	−0.46**
6. Workplace alienation	−0.41**	−0.39**	−0.40**	−0.41**	−0.46**	1.00

** *p* <0.01.

### 3.5 Predictors of Workplace Alienation

To identify predictors of workplace alienation, preliminary bivariate analyses were conducted using independent samples t‐tests, one‐way ANOVA, and Pearson correlation. These analyses determined which sociodemographic and work‐related variables showed significant associations with workplace alienation and were thus eligible for inclusion in hierarchical regression.

An independent samples *t*‐test was performed to assess differences in workplace alienation across gender, educational level, and marital status. Gender was categorized as male (*n* = 112) and female (*n* = 71), educational level as bachelor's (*n* = 143) and master's (*n* = 40), and marital status as married (*n* = 152) and single (*n* = 31). None of the comparisons yielded statistically significant differences (Table 3).

**Table 3:** Independent samples *T*‐test (*N* = 183).

Variable	Category	Mean	SD	*t*‐value	*p* value
Gender	Male	2.74	0.46	1.021	0.308
	Female	2.67	0.49		
Educational level	Bachelor's	2.73	0.46	0.871	0.385
	Master's	2.66	0.53		
Marital status	Married	2.71	0.46	−0.552	0.581
	Single	2.76	0.51		

A one‐way ANOVA was conducted to examine whether there were statistically significant differences in workplace alienation based on categorical variables with three or more levels, specifically work area/unit and years of nursing experience. The results indicated no statistically significant differences in workplace alienation by work area/unit (*F* = 0.53, *p* = 0.79) or by years of experience (*F* = 0.63, *p* = 0.60), Table 4.

**Table 4:** One‐way ANOVA results.

Variable	Category	Mean	SD	*F* value	*p* value
Work area or unit	Medical/Surgical departments	2.72	0.40	0.53	0.79
	Emergency department	2.78	0.40		
	Critical care departments	2,59	0.49		
	OB/GYN departments	2.68	0.51		
	Pediatric departments⁠	2.68	0.61		
	Outpatient departments	2.71	0.54		
	Others	2.76	0.49		
Years of experience	Less than 5 years	2.67	0.50	0.63	0.60
	5 to less than 10 years	2.74	0.47		
	10 to less than 15 years	2.77	0.41		
	15 years and older	2.65	0.51		

Pearson Correlation analysis was used to determine the extent of a statistically significant relationship between age and workplace alienation. The results of the analysis revealed no statistically significant relationship between age and workplace alienation (*r* = −0.02, *p* = 0.81). The correlation was close to zero, indicating no meaningful linear relationship between nurses' age and their workplace‐alienation scores in this sample.

Because none of the previous results were statistically significant in the three tests for personal, occupational, and work‐related variables, hierarchical regression was not used. Instead, simple linear regression was used to determine the extent of a statistically significant relationship between perceived organizational justice and workplace alienation. The perceived organizational justice variable explained approximately 20.7% of the variance in workplace alienation. Simple linear regression indicated that perceived organizational justice was significantly and inversely associated with workplace alienation (β=−0.46, $t_{181}=-6.88$, p<0.001; Table 5). This indicates that nurses reporting greater organizational justice tended to report lower workplace alienation. Perceived organizational justice explained 21% of the variability in workplace‐alienation scores ($R^{2}=0.21$). Because the study was cross‐sectional, this association should not be interpreted as causal (Table 5).

**Table 5:** Simple linear regression examining the association between perceived organizational justice and workplace alienation.

Independent variable	*t*	*t* Sig.	*R*	*R* ^2^	*F*	*p* value
Perceived organizational justice	−6.88	< 0.01	0.46	0.21	47.29	< 0.01

## 4 Discussion

This study demonstrates a significant inverse relationship between perceived organizational justice and workplace alienation among nurses in government hospitals of Northern Saudi Arabia. High perceptions of fairness directly correspond to reduced feelings of disconnection and isolation at work, offering actionable insights for healthcare improvement. These patterns align with social exchange theory, where fairness fosters trust and reciprocity, mitigating estrangement [19].

This inverse justice‐alienation relationship corroborates regional findings from Awwad and Adaileh [16‏ and Abd‐Elrhaman et al. (2020), who reported comparable correlations (*r* = −0.40 to −0.50) among Middle Eastern healthcare workers. Distributive justice's leading role mirrors Zahran and Eldossoqi [17], where equitable outcomes reduced burnout and meaninglessness in high‐stress clinical environments. The elevated Saudi justice perceptions surpass those in Abou Hashish et al. [10] among Egyptian nurses, likely attributable to Vision 2030 reforms that strengthened Ministry of Health transparency and accountability [13].

Internationally [12], replicate these patterns through self‐determination theory, with justice satisfying autonomy and relatedness needs [20]. Non‐significant sociodemographics diverge from Song et al. (2024), potentially due to sample homogeneity and cultural duty emphasis [16]. The generally low alienation than previous research could reflect Saudi retention incentives. Unit parity, including emergency nurses, extends Colquitt et al.'s (2023) evidence of justice's broad protective effects against errors and turnover [21].

### 4.1 Strengths and Limitations

This study offers several strengths as the first examination of the justice‐alienation nexus in Northern Saudi Arabia during Vision 2030 transformations, using validated instruments. However, limitations include the cross‐sectional design's inability to establish causality, self‐report social desirability risks in hierarchical settings [21], convenience sampling restricting generalizability to Al‐Jouf government hospitals (excluding private facilities, expatriates, and other regions), and absence of objective outcomes like turnover rates. Another limitation is that the study focused on organizational justice as the primary organizational correlate of workplace alienation and did not assess other potentially relevant determinants, such as leadership, organizational climate, workload, autonomy, organizational support, role ambiguity, or emotional exhaustion. Consequently, the findings should not be interpreted as indicating that organizational justice explains workplace alienation independently of other organizational and occupational factors. Future research should use multivariable and longitudinal designs incorporating a broader range of organizational and individual factors to provide a more comprehensive understanding of workplace alienation among nurses.

### 4.2 Implications and Recommendations

Healthcare administrators should prioritize interactional justice through targeted communication and respect training programs, alongside transparent reward systems, equitable workloads, and performance recognition to address distributive concerns. Procedural empowerment may be achieved through nurse decision‐making committees to fight powerlessness and mentorship and mental health assistance specific to novice nurses. To align with the Health Sector Transformation Program goals (2021) and Vision 2030 workforce goals, policymakers are advised to integrate metrics of justice in the Ministry of Health reviews and nursing education and require standardized training in fairness.

Further studies need to seek longitudinal intervention research, e.g., randomized controlled studies of justice training, in greater geographical regions of Saudi Arabia including expatriates and the private sector to prove causality. The qualitative methods would help to explain dimension‐specific processes (e.g., distributive justice), and comparative studies with the GCC countries would have increased the generalizability.

## 5 Conclusion

Perceived organizational justice was associated with lower workplace alienation and explained 21% of its variance among nurses in Northern Saudi Arabia. Fair procedures, equitable rewards, respectful treatment, and clear communication may support nurses' well‐being, trust, and engagement.

Hospital leaders should strengthen organizational justice through transparent rewards, effective communication, nurse participation in decision‐making, and support for early‐career nurses. These actions may improve nurse retention, job satisfaction, and quality of care. Future longitudinal, multi‐regional studies should examine whether these associations are sustained across Saudi Arabia and the Gulf region.

## Author Contributions

**Ahmad Rayan:** conceptualization, methodology, formal analysis, supervision, writing – review and editing. **Mamdooh Arrowaili:** conceptualization, methodology, formal analysis, writing – review and editing. **Manal Hassan Baqeas:** conceptualization, methodology, writing – review and editing. **Fadwa Alhalaiqa:** conceptualization, methodology, writing – review and editing. **Suhair Al‐Ghabeesh:** conceptualization, methodology, writing – review and editing. **Hanan Alyami:** conceptualization, methodology, writing – review and editing. **Mirna Fawaz:** conceptualization, methodology, writing – review and editing.

## AI‐Assisted Language Editing Disclosure

Grammarly was used solely to support grammar, spelling, and language editing. It was not used to generate scientific content, analyze data, interpret results, or make scholarly decisions. All authors reviewed and revised the manuscript and accept full responsibility for its content.

## Ethics Statement

The study was approved by the Institutional Review Board at Zarqa University, adhering to ethical standards in research.

## Conflicts of Interest

The authors declare no conflicts of interest.

## Transparency Statement

Mirna Fawaz, the manuscript guarantor, affirms that this manuscript is an honest, accurate, and transparent account of the study being reported; that no important aspects of the study have been omitted; and that any discrepancies from the study as planned have been explained.

## Data Availability

The data that support the findings of this study are available from the corresponding author upon reasonable request.
